# Women Doctors

**Published:** 1920-10-09

**Authors:** 


### Women Doctors.

### THE PROBLEM FOR THE MEDICAL SCHOOLS.

A month or two ago we published two letters for and against women doctors, but, whatever views may be held upon the subject, there is no denying the fact that women doctors have come to stay, and in increasing numbers. One of our correspondents questioned the temperamental qualifications of women; a writer in the *Manchester Guardian* claims emphatically that experience has disposed of such doubts. Women who have qualified and have assumed duties of house surgeons at hospitals have proved that in physical endurance, knack in surgical practice, tact in dealing with patients, and judgment in emergencies they rival men doctors. At the Royal Free Hospital there are six resident women medical officers, and only one man, and the women deal with every kind of casualty case that comes in.

This same correspondent considers the present problem created for the London medical schools by the increasing number of women students. He says that during the war the experiment of admitting women to medicine became an obviously practical thing. London University in 1917 opened the doors of the medical schools to women. There had, of course, always been the London (Royal Free Hospital) School of Medicine for Women, which had been steadily training women, and women only, for the profession; but now the experiment was extended to the hospital medical schools of University College, King's College, London Hospital, Charing Cross, Westminster, and St. Mary's.

For a year St. George's also admitted women, but women are no longer admitted there. At the other medical schools in London, including such well-known names as Bart's, Guy's, the Middlesex, and St. Thomas's, women are still excluded.

Now that the men medicals have returned from the war, and the normal supply of men medicals each year has reasserted itself in addition, an embarrassing situation is created by the number of women medicals, who by now have completed as many as three years of their five-years' course. At the London School of Medicine for Women alone there are now 450 women students, nearly 100 of whom are new this term. The medical schools cannot possibly take them all. To reject the women would reflect upon the authorities as a graceless piece of opportunism no less than a piece of bad judgment.

